# Shaping Up the Tumor Microenvironment With Cellular Fibronectin

**DOI:** 10.3389/fonc.2020.00641

**Published:** 2020-04-30

**Authors:** Georgios Efthymiou, Angélique Saint, Michaël Ruff, Zeinab Rekad, Delphine Ciais, Ellen Van Obberghen-Schilling

**Affiliations:** ^1^Université Côte d'Azur, CNRS, INSERM, iBV, Nice, France; ^2^Centre Antoine Lacassagne, Nice, France

**Keywords:** tumor, extracellular matrix, fibronectin, oncofetal variants, cancer hallmarks

## Abstract

Normal tissue homeostasis and architecture restrain tumor growth. Thus, for a tumor to develop and spread, malignant cells must overcome growth-repressive inputs from surrounding tissue and escape immune surveillance mechanisms that curb cancer progression. This is achieved by promoting the conversion of a physiological microenvironment to a pro-tumoral state and it requires a constant dialog between malignant cells and ostensibly normal cells of adjacent tissue. Pro-tumoral reprogramming of the stroma is accompanied by an upregulation of certain extracellular matrix (ECM) proteins and their cognate receptors. Fibronectin (FN) is one such component of the tumor matrisome. This large multidomain glycoprotein dimer expressed over a wide range of human cancers is assembled by cell-driven forces into a fibrillar array that provides an obligate scaffold for the deposition of other matrix proteins and binding sites for functionalization by soluble factors in the tumor microenvironment. Encoded by a single gene, FN regulates the proliferation, motile behavior and fate of multiple cell types, largely through mechanisms that involve integrin-mediated signaling. These processes are coordinated by distinct isoforms of FN, collectively known as cellular FN (as opposed to circulating plasma FN) that arise through alternative splicing of the *FN1* gene. Cellular FN isoforms differ in their solubility, receptor binding ability and spatiotemporal expression, and functions that have yet to be fully defined. FN induction at tumor sites constitutes an important step in the acquisition of biological capabilities required for several cancer hallmarks such as sustaining proliferative signaling, promoting angiogenesis, facilitating invasion and metastasis, modulating growth suppressor activity and regulating anti-tumoral immunity. In this review, we will first provide an overview of ECM reprogramming through tumor-stroma crosstalk, then focus on the role of cellular FN in tumor progression with respect to these hallmarks. Last, we will discuss the impact of dysregulated ECM on clinical efficacy of classical (radio-/chemo-) therapies and emerging treatments that target immune checkpoints and explore how our expanding knowledge of the tumor ECM and the central role of FN can be leveraged for therapeutic benefit.

## Introduction

**Figure 1:**
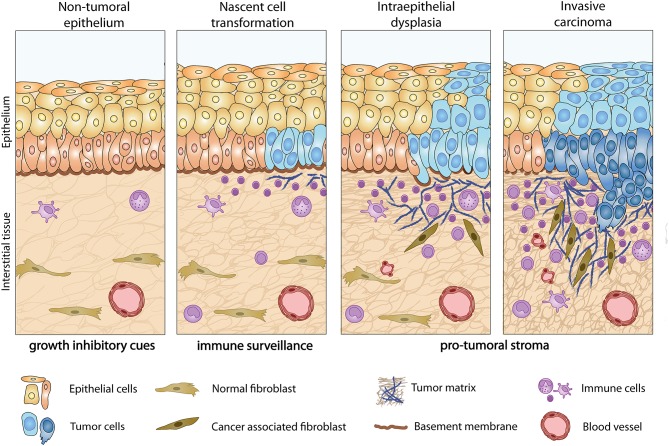
Tumor-induced stromagenic reprogramming during carcinoma progression. In normal epithelial tissue (left) homeostatic processes and the presence of an intact basement membrane restrain tumor growth. During early stages of tumor development, nascently transformed cells release pro-inflammatory cues that recruit immune cells to the dermal-epidermal interface and stimulate a wound healing response characterized by fibroblast activation and recruitment of angiogenic blood vessels. Activated stromal cells in turn promote the invasive phenotype of tumor cells through direct and indirect mechanisms. This tumor-stroma interplay is accompanied by the upregulation of a specific set of ECM components (1) and their receptors (2), in both tumor and stromal cells.

Historically, studies addressing the genesis and progression of cancer have focused on the genotype of tumor cells. In the case of carcinomas, nascently transformed epithelial cells progress to an invasive phenotype by the accumulation of mutations. However, this is only part of the story, as schematized in Figure 1. Tumor progression and expansion are accompanied by major changes in the tissue immediately adjacent to premalignant lesions and require reciprocal interactions between malignant epithelial cells and stromal cells. Tumor-induced alterations in the reactive stroma involving modifications in ECM composition, organization, and physical properties, have drawn increasing attention over the past few years as a more holistic view of tumor progression, complexity and heterogeneity of tumor microenvironment (TME) is being embraced and scrutinized for the discovery of novel, clinically relevant therapeutic opportunities.

The ECM proteins induced in tumor tissue are often development- and disease-specific isoforms generated by alternative splicing events. Such is the case with fibronectin (FN), as described below. Before focusing on FN and its multi-faceted role in the tumor setting, we will briefly discuss important notions and emerging themes regarding the production, organization and remodeling of ECM in tumor tissue. Numerous outstanding reviews are cited in this section to provide a more comprehensive picture of these themes.

## Stromal Reprogramming Through Tumor-Stroma Crosstalk

### Tumor Matrisome

Gene expression screens have revealed that many genes encoding ECM components are dysregulated during tumor progression (3, 4). As the ECM is composed of large insoluble components, its protein composition has been detailed only recently. In an effort led by the laboratory of Richard Hynes, proteomics-based methods coupled with bioinformatics were used to define the “matrisome” of several normal and diseased tissues, including multiple tumor types (5). The computationally predicted matrisome corresponds to over 1,000 genes encoding a set of 278 core components and 753 matrisome-associated proteins, of which 86% of the core matrisome proteins and 58% of the matrisome-associated components have been detected in tissues using ECM-focused proteomics strategies [see (6) for the latest Matrisome database]. Examples of upregulated ECM components in cancer include collagens, non-collagen glycoproteins (FN, tenascin C, periostin), proteoglycans (biglycan, decorin), ECM regulators (cathepsin B, LOXL), and secreted factors (TGF-β1) [reviewed in (7)], to name only a few.

### Source of Tumor ECM

**Figure 2:**
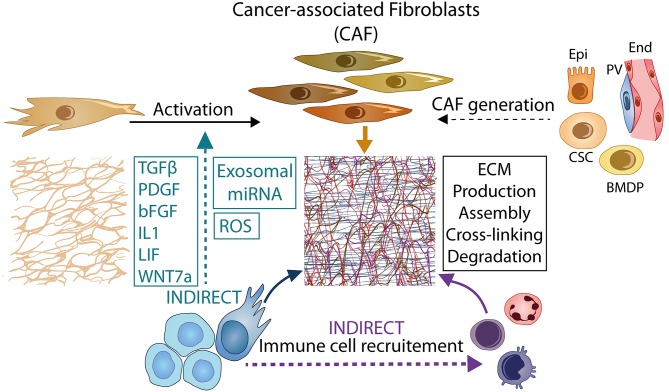
Tumor-CAF crosstalk and molecular mediators of ECM reprogramming. Tumor cells promote the generation of CAFs from resident fibroblasts or cells of different origin through the secretion of cytokines (e.g., TGF-β, PDGF, bFGF, IL1, LIF, WNT7A), the production of ROS, and exosomal delivery of miRNA. CAFs remodel the ECM by producing, assembling, cross-linking, and degrading ECM components. Tumor cells and infiltrating immune cells are also important proponents of ECM remodeling. The complex crosstalk between tumor and stromal cells leads to a global increase in ECM abundance and stiffness which in turn amplifies CAF activation via a positive feedback loop. Epi, epithelial cells; End, endothelial cells; PV, peri-vascular cells; CSC, cancer stem cells; BMDP, bone marrow derived precursor cells.

Fibroblasts are considered to be the major source of ECM in tumor tissue as they are abundant, highly secretory and competent for ECM assembly [reviewed in (8)]. Other stromal cells, including vascular and immune cells, contribute to tumor ECM production as well. *In vitro* and *in vivo* studies have established that matrix proteins expressed by malignant cells also become directly incorporated in the matrix. Sets of tumor cell-derived ECM proteins were elegantly identified using xenograft models in which human tumor cells were grafted in murine hosts (5, 9–11). Interestingly, in these models the ECM composition was found to differ depending on the metastatic potential of the malignant cells, their tissue of origin, and whether they were derived from primary tumors or metastases. The multicellular origin of the neoplastic ECM holds true for human tumors as well. In a single-cell transcriptomic analysis of oral squamous cell carcinomas, ECM genes that are often linked with EMT (e.g., TGFBI, LAMC2, tenascin C) were found to be upregulated in carcinoma cells. Interestingly, their expression was enhanced in a subset of tumor cells displaying a partial EMT phenotype and located in close apposition to surrounding stroma, as determined by immunohistochemistry (12). These results indicate that paracrine signals from the stromal compartment trigger ECM gene expression in leading-edge cancer cells and they suggest a role for the upregulated matrix proteins in tumor invasion. Just as stromal mediators can trigger ECM gene expression in malignant cells, malignant cells can increase matrix production in the stromal compartment by promoting the activation of normal fibroblasts, of various origins, to carcinoma-associated fibroblasts (CAFs) as schematized in Figure 2. In addition to reprogramming CAF precursors, cancer cells recruit immune cells to the TME, such as tumor-associated macrophages (TAMs), neutrophils, dendritic cells, natural killer cells, T and B lymphocytes (13, 14). All of these cells represent a potential source of ECM components. This has been shown for TAMs which are extremely abundant in several tumor pathologies [as reviewed in (15, 16)]. TAMs also “enrich” the tumor matrix by secreting high levels of ECM-binding cytokines and growth factors that stimulate fibroblast activation (17).

### CAF Heterogeneity

The tumor-promoting effects of CAFs have been widely investigated and include the enhancement of cell proliferation, survival, migration/invasion, angiogenesis, chemoresistance, and immunosuppression, as detailed in recent reviews (18–20). Their activity is mediated through the secretion of a plethora of growth factors, cytokines and exosomes, but also through the production and remodeling of the ECM. CAFs have been equated to myofibroblasts, or activated fibroblasts linked to wound healing and contracture (21), because they often express α-smooth muscle actin (αSMA). However, it is now clear that CAFs exist as a heterogeneous population with distinct, yet overlapping, functions. Precise characterization of CAFs has been difficult as no marker is exclusive or absolute. Various combinations of markers including but not limited to αSMA, FAP, FSP1, CAV1, IL6, VIM, ITGB1, PDGFRα/β have been used to identify CAF subtypes in different tumor tissues using flow cytometry (22, 23) and single cell transcriptomics (12, 24). However, the distinct functions of these CAF subtypes and their associated ECM are not well-characterized to date.

CAFs arise from several different cell types including resident fibroblasts, bone marrow-derived precursor cells, vascular smooth muscle cells, pericytes, cancer stem cells, as well as endothelial and certain epithelial cells via endothelial- and epithelial-to-mesenchymal transition, respectively (25–28). CAF heterogeneity is thus due in part to their diverse origin, which is still under intense investigation and undoubtedly depends on the tumor (sub)type and anatomical localization. In addition, extracellular signals from the microenvironment, in particular mediators from other cells, drive CAF heterogeneity and dynamic changes in biomarker expression. Moreover, the positioning of CAFs in time and space, with respect to tumor cells, is an important determinant underlying the generation of CAF subtypes (23, 29–31).

### CAF Generation

Until recently, the activation states of CAFs have been oversimplified and reduced to normal fibroblasts and activated fibroblasts, determined by αSMA expression. However, as indicated above, the activation state of CAFs cannot be solely defined by αSMA expression since certain CAF populations display only minimal αSMA levels (30, 32, 33).

Most inducers of CAF-like phenotypes (Figure 2) are also involved in fibroblast conversion to myofibroblasts during fibrosis, such as TGF-β. TGF-β is a master regulator of myofibroblast and CAF generation (34, 35) and a powerful inducer of several ECM components including collagen of type I, II, III, IV, and V, FN, thrombospondin, osteopontin, tenascin C, TGFBI, periostin, elastin, hyaluronic acid, osteonectin/SPARC, as well as chondroitin/dermatan sulfate proteoglycans, such as biglycan and decorin (36–38). TGF-β not only activates resident fibroblasts but also promotes the differentiation of CAF precursors including adipose tissue-derived stem cells, endothelial cells, and bone marrow-derived mesenchymal stem cells (39).

Similar to TGF-β, platelet-derived growth factor (PDGF) and fibroblast growth factor 2 (bFGF/FGF2) play critical roles in myofibroblast activation and fibrosis (40–42). In cancer, they were found to regulate CAF activation and αSMA expression as well, although their effects varied depending on the cell types examined (32, 43–46).

Reactive oxygen species (ROS) generated by cancer cells were shown to promote the fibroblast-to-myofibroblast transition by a mechanism involving TGF-β, PDGF, and CXCL12 signaling (47). Certain cytokines are important activators of fibroblasts, such as IL1α, which triggers fibroblast differentiation in inflammatory CAFs by inducing LIF, a cytokine of the IL6 family that activates JAK/STAT signaling (29). Apart from growth factors and cytokines, cancer cells also produce extracellular vesicles containing miRNA (e.g., miR-9, miR-155, miR-211) and proteins (TGF-β, BMP, and tetraspanins) that induce fibroblast activation or CAF generation from mesenchymal stem cells [reviewed in (48)].

### ECM Organization and Remodeling

In addition to the stimulation of ECM protein expression, tumor-fibroblast crosstalk profoundly impacts matrix assembly, cross-linking and remodeling. The assembly of matrix macromolecules into a 3D structure is a dynamic process largely carried out in the tumor stroma by CAFs. Fibrillar collagens are the major components of the tumor ECM and collagen architecture is severely altered in tumor tissue [see (49, 50)]. In breast cancer, distinct patterns of fibrillar collagen organization, termed “tumor-associated collagen signatures” (TACS 1–3), have been defined to classify the changes in collagen arrangement that accompany carcinoma progression (51). TACS 3, characterized by straightened and aligned collagen fibers oriented perpendicular to the tumor boundary, was found to be an independent prognostic indicator of poor survival (52). For the present review, it is important to note the interdependence of collagen and FN networks. Fibrillar assembly of FN is required for collagen fibrillogenesis (53–56). Indeed, FN is a provisional matrix molecule (57) that provides a template for deposition of not only collagen, but for several ECM components including LTBP1, fibulin, and thrombospondin [(58) and references therein] as well.

## Cellular “Oncofetal” Fibronectin: A Key Multi-Regulatory Component of the Tumor ECM

FN is a major core component of the tumor matrisome. Initially discovered as a “contaminant” in one of the steps of fibrinogen isolation more than 70 years ago (59), it is now one of the most extensively studied proteins, in terms of structural analysis and functional aspects.

### Fibronectin Structure

**Figure 3:**
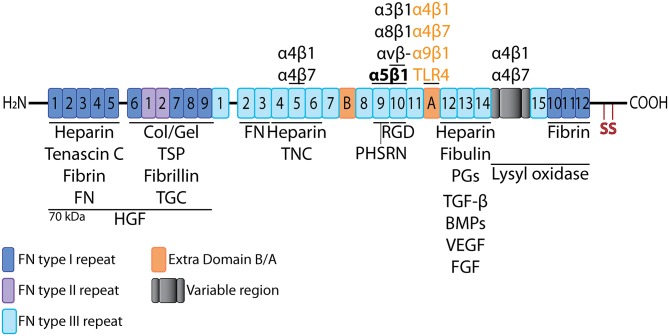
Fibronectin linear structure. Schematic representation of the linear structure of FN molecule, showing the different types of repeats and multiple binding sites for cells and other molecules. Adapted from Xu and Mosher (62) and Van Obberghen-Schilling et al. (63). TSP, thrombospondin; Col/Gel, collagen/gelatin; PGs, proteoglycans; TGC, tissue transglutaminase.

FN is a high molecular weight glycoprotein composed of two similar subunits of 220–250 kDa (60, 61), linked together via disulfide bonding between two carboxy-terminal cysteine residues per subunit. FN is secreted in a soluble form by hepatocytes into the bloodstream (plasma FN, pFN), or expressed in tissues by fibroblasts and other cell types (cellular FN, cFN) forming an insoluble mesh. The primary structure of a FN subunit is characterized by the presence of three distinct types of repeats [reviewed in (62)], as schematized in Figure 3. There are 12 type I repeats (FNI_1−12_), two type II repeats (FNII_1−2_), and 15 (up to 17, see below) type III repeats [FNIII_1−15_ (64)]. Apart from the repetitive domains, there is also a variable region (V or IIICS, type III connecting segment) that lies between FNIII_14_ and FNIII_15_. The V region is not addressed in this review. The reader is invited to see other works, such as Xu et al. (62), Schwarzbauer and DeSimone (65), and references therein.

FNI and FNII repeats are composed of 45 and 60 amino acids, respectively, and they contain cysteine residues that form intra-domain disulfide bonds (66, 67). By contrast, FNIII repeats are composed of 90 amino acids, they contain no cysteines, and are organized in two antiparallel β-sheets folded in a sandwich-like conformation with a hydrophobic core (68–71). This structure results in a compact form that can be extended when strain is applied (72). There are 15 FNIII domains present in every FN monomer, and two additional domains termed Extra Domains (EDB and EDA) that are only included in cFN via alternative splicing, as described below.

### Fibronectin Interactions and Function

The modular structure of FN and its multiple post-translational modifications result in numerous interactions with a variety of molecules that mediate cell attachment, ECM assembly, cell motility, cytoskeleton contractility, and host-pathogen interactions, to name just a few. Integrins represent the major family of cellular receptors through which FN exerts multiple functions in health and disease (73).

Integrin α5β1 is the “classic” FN receptor, that recognizes the tripeptide cell-binding site Arg-Gly-Asp (RGD) located in the FNIII_10_ repeat (71, 74–76). This interaction is facilitated and further stabilized by the synergistic effect of the PHSRN site located in FNIII_9_ (75). Binding of FN to α5β1 results in activation of the integrin, subsequently leading in Rho-mediated acto-myosin contractility that in turn promotes assembly of fibronectin into a fibrillar matrix (77–80). Integrin αvβ3 also binds the RGD site, as do α3β1, α8β1, αIIbβ3, and other αv-based integrins [reviewed in (62)], while α4β1 and α4β7 bind to specific sequences in FNIII_5_ (81), FNIII_14_ (76), and in the V region (82–84).

Apart from cell receptors, FN also interacts with ECM components via distinct sites in the FN molecule. Through the 70 kDa region (see Figure 3), FN binds collagen and gelatin, as well as fibrillin, and thrombospondin (85). This results in the enrichment of the provisional FN mesh with additional components of the matrisome, contributing to ECM maturation, which in turn promotes cell adhesion *in vivo*, and blood vessel morphogenesis during embryonic development and pathological angiogenesis [reviewed in (62, 63)]. The formation of the provisional meshwork lies on the ability of FN to self-associate at three distinct regions [reviewed in (86)], promoting polymeric assembly and mediating FN fibrillogenesis (87).

FN fibrillogenesis is a multistep process that involves the modular structure of FN, interactions of FN with other molecules, and cytoskeletal rearrangements in cells that assemble it [reviewed in (80)]. In brief, FN in a compact conformation is presented to the cell surface in an autocrine or paracrine manner. FN binding to α5β1 triggers integrin activation, clustering and the recruitment of cytoplasmic partners, including ILK, PINCH, parvin, and tensin. This intracellular machinery drives Rho-mediated stress fiber formation. Cell-generated acto-myosin contractility applies strain on the FN molecule resulting in its switch from a compact to a stretched state, thereby allowing intermolecular interactions required for FN incorporation into fibrils (72, 88, 89). Furthermore, integrin clustering and formation of complexes with additional cell receptors, like syndecan-4 [reviewed in (90)] or urokinase plasminogen activator receptor [uPAR (91)], can enhance FN assembly and strengthen FN-integrin binding (91–93). Finally, longitudinal and lateral association of FN molecules to existing fibrils results in FN polymerization, probably mediated by the protein-disulfide isomerase activity of FN, located in the FNI_12_ (94).

On this polymerized FN network, many other ECM associated components are assembled, such as heparin sulfate proteoglycans via their respective binding sites (64, 95–98), enhancing adhesion and spreading (92, 99). Similarly, TNC binds to FN and fine tunes cell adhesion and motility during angiogenesis and tumor progression [reviewed in (63)]. Finally, FN acts as a scaffold upon which the bioavailability and activity of several growth factors is orchestrated (100). Interaction of FN with growth factors (e.g., members of the TGF-β superfamily, PDGF, HGF, VEGF, FGF) may impact cell migration, cell proliferation, survival signals, and angiogenesis, as downstream outcomes of their activation through mechanical or enzymatic activation (101).

### Fibronectin Splicing: The Oncofetal Fibronectin Variants

The 75 kbp long human *FN1* gene is composed of 46 exons, and produces up to 20 distinct isoforms via alternative splicing [(102) and reviewed in (76)]. The first alternatively spliced region identified was the Extra Domain A (EDA, EIIIA, EDI), a FNIII repeat lying between FNIII_11_ and FNIII_12_, followed by the discovery of Extra Domain B (EDB, EIIIB, EDII) between FNIII_7_ and FNIII_8_ (103–106). Extra Domains are encoded by a single exon each, and they are only present in the cFN. Conversely, pFN lacks both Extra Domains.

Regulation of FN splicing depends strictly on tissue type and developmental stage, and it is tightly coupled to the activity of members of the SR protein family [(e.g., SRSF3, SRSF5) reviewed in (107)]. pFN is expressed throughout the entire lifespan of the organism, though declining with age (108–110). In contrast, cFN expression is elevated during embryonic development but diminishes significantly after birth (111–113). Intriguingly, cFN is re-expressed during the adult life under certain conditions that involve TGF-β signaling. Such conditions include tissue repair, fibrosis, angiogenesis, and cancer (114–117). Accordingly, increased SRSF3 and SRSF5 expression correlates with certain types of cancers [i.e., oral squamous cell carcinoma (118, 119)] and TGF-β signaling has been shown to regulate their expression (120), similar to that of cFNs (121–123). In light of the restricted expression of Extra Domain-containing FN, the hypernym “oncofetal FN” was used in the early 1980s to collectively describe these FN variants.

### Functional Roles of the Extra Domains

Despite extensive research, the precise functional properties of EDB and EDA have yet to be fully deciphered. Non-exhaustive lists of *in vitro* and *in vivo* studies regarding EDB and EDA functions can be found in Muro et al. (124) and To and Midwood (125).

A series of elegant approaches have shed light in the functions of EDB and EDA. In two independent *in vivo* studies, mice expressing FN with constitutively included or excluded EDA were generated. All animals were viable and developed normally. However, mice lacking EDA displayed abnormal and delayed skin wound healing, and decreased motor coordination abilities, while mice constitutively expressing EDA showed a pronounced decrease in the level of FN in all tissues and decreased locomotory activity (126, 127). Interestingly, both mouse strains had shorter lifespans compared to control littermates (126). By contrast, deletion of EDB displayed no significant phenotype in mouse development and fertility, but fibroblasts extracted from EDB-null mice grew more slowly *in vitro*, and were less efficient at depositing and assembling FN (128). Most importantly, absence of both Extra Domains was deleterious for the organism due to severe cardiovascular defects (e.g., vascular leakage, defective angiogenesis) (129) suggesting that the Extra Domains have overlapping functions during embryonic development, and at least one of the two is necessary for normal body growth.

In disease-challenged situations in the adult, when cFN expression reappears, both Extra Domains have been correlated with a pro-fibrotic tissue landscape. More specifically, increased expression of FN-EDA resulted in differentiation of normal lipocytes to myofibroblast-like cells (115) and this phenotype was mediated by TGF-β1 (130). Furthermore, absence of EDA-containing FN in an idiopathic pulmonary lung fibrosis mouse model resulted in less collagen deposition and fewer α-SMA expressing myofibroblasts. This effect correlated with diminished activation of TGF-β suggesting that EDA is implicated in latent TGF-β activation (124). Most importantly, the presence of EDA highly correlated with enhanced matrix remodeling, matrix metalloproteinase (MMP) expression, and re-organization of the actin cytoskeleton (131), pointing toward a pro-fibrotic role for EDA. Similar findings were obtained in a tumoral context when tumor sections were found to be enriched in FN-EDB and FN-EDA in newly formed blood vessels of the tumor (132, 133). Reinforcing the potential tumorigenic role of cFN, EDA-containing FN induced G1-S phase transition by increasing the expression of Cyclin D1 and upregulation of integrin-mediated mitogenic signal transduction (134).

The aforementioned effects may be due in part to the increased cell receptor repertoire of cFN compared to pFN. More specifically, EDA contains an EDHIGEL sequence that has been identified as a binding site for integrins α4β1, α4β7, and α9β1 (135, 136). Furthermore, EDA is a ligand and activator of toll-like receptor 4 (TLR4) (137), thus triggering immune responses, described in subsequent sections. Conversely, no receptor has been identified so far for EDB, though a role has been suggested for EDB in osteoblast differentiation involving a β1-containing integrin (138).

## cFN and the Hallmarks of Cancer

**Figure 4:**
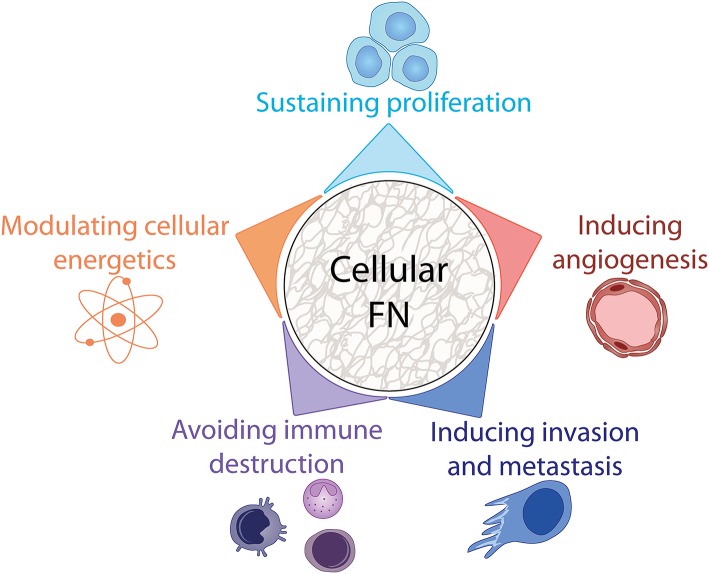
Involvement of cFN in cancer hallmarks. FN participates in tumor progression by impacting several enabling hallmarks of cancer, see text for details.

A major finding during the early days of FN research was that surface fibroblast antigen (SFA), as FN was named at the time, was significantly reduced in quantity upon malignant transformation of chicken and human fibroblasts infected with Rous Sarcoma Virus (RSV) (139, 140). However, it is now widely acknowledged that FN is strongly upregulated in several different tumor types. As the “malignant cell-centric” view of tumors shifted in recent years to a more inclusive view that encompasses their microenvironment, reports of the tumor suppressive functions of FN have been replaced by reports of its positive role in tumor growth and metastasis [reviewed in (86)]. This can be at least partly explained by the role of FN as a provisional matrix component promoting the formation of a primed TME that sustains cancer cell survival, stimulates proliferation, migration, angiogenesis and immune modulation (Figure 4). In this section, we will comment on the implication of cFN in these processes and highlight how cFN induction at tumor sites regulates various cellular responses that characterize the cancer hallmarks defined by Hanahan and Weinberg in 2001 (141) and amended in 2011 (142).

### Sustaining Proliferative Signaling and Evading Growth Suppression

Several lines of evidence have brought FN under the spotlight for its role in cell proliferation. Developmental processes, such as the establishment of antero-posterior polarity, the formation of the neural tube and mesodermally derived tissues are thought to be regulated by FN-mediated cell proliferation (143). Regarding tissue homeostasis, FN choreographs the proliferative phase of wound healing by bringing together different cells and components (144). In cancer, FN is a basic component of the tumor niche that has been shown to facilitate cancer cell proliferation and survival. *In vitro* studies have underlined the role of FN in promoting cancer cell growth, survival, and invasion in glioma (145), renal cell carcinoma (146), and gall bladder carcinoma (147). *In vivo*, it was shown that tumor cells injected in mice lacking circulating FN grew more slowly and apoptosis was increased (148). Similarly, tissue-specific depletion of FN resulted in lower tumor cell proliferation and invasion in the bone marrow (148).

One of the earliest studies on cFN variants highlighted the potency of EDA-containing FN to induce expression of cyclin D1, hyperphosphorylation of pRb and activation of ERK2 resulting in cell cycle progression (134). Similar results were obtained 10 years later when Kohan and colleagues described that recombinant EDA-containing peptides were able to induce MAPK-ERK1/2 activation and fibroblast differentiation through α4β7 binding and FAK activation (149). In a 3D cell culture system, blocking of FN-α5β1 interaction induced apoptosis in breast cancer cells via a mechanism that involves Akt, suggesting a protective role of FN for tumor cells (150). Though the authors did not directly assess the anti-apoptotic role of EDA *per se*, they hypothesized that it is the EDA-mediated strengthening of FN-α5β1 interactions (151) that results in the protective effect of FN against cell death. Finally, in two human tumor cell lines it was shown utilizing CRISPR/Cas9 technology that exclusion of EDA resulted in a pronounced decrease in cell proliferation (152).

*In vivo*, ovarian cancer cells displayed decreased proliferation and metastasis in mice bearing a tissue-specific deletion of *Fn1* in the lining of the peritoneal cavity. The effects were attributed to a tumor-stroma crosstalk and the participation of TGF-β signaling (153). The splicing pattern of the FN produced in control mice was not identified, but given its cellular nature, and the implication of TGF-β in Extra Domain inclusion (see previous section), addressing how normal cells residing in the TME influence tumor cells by cFN expression is a question worth-addressing.

In contrast to EDA, the role of EDB in tumor cell proliferation is largely unknown, in spite of its increased presence in the TME. In vascular endothelial cells, EDB-containing peptides were found to stimulate proliferation (154), while EDB knock-down impaired cell growth (154, 155).

### Inducing Angiogenesis

FN clearly occupies a central note in the “angiome,” the global protein connectivity network of genes associated with angiogenesis (156). The importance of FN in angiogenesis was first revealed by genetic studies in the mouse demonstrating that invalidation of the FN gene induces embryonic lethality (around E9.5) with cardiovascular and angiogenesis defects (143). Intriguingly, specific ablation of cFN (including both EDB and EDA domains) in mice that still express pFN also triggered defective angiogenesis leading to hemorrhagic vessels and embryonic lethality at E10.5 (129), attesting to a critical role for these cFN exons in developmental angiogenesis. The source of the cFN is also critical for its role in vascular development. In the neonatal retina, angiogenesis is regulated by endothelium-derived FN in an autocrine manner (157). This is an important notion, as FN production in endothelial cells is tightly coupled to its assembly (155), and assembly of cFN into a three-dimensional fibrillar meshwork is essential for neovessel formation (158).

Concerning the role of FN in tumor angiogenesis, results from animal models (e.g., inducible deletion, tumor xenografts) are less clear. Post-natal deletion of endothelial cFN in a spontaneous RIPTag-driven model of carcinogenesis fails to inhibit tumor angiogenesis (159, 160), suggesting a complex functional role of FN in tumor angiogenesis and partially explaining the disappointing results of targeting FN-binding integrins in the clinic, as discussed below. Nonetheless, cFN has been recognized for quite some time to be a useful marker of cancer-associated vessels (133, 161, 162). Expression of cFN is also upregulated in malignant cells of certain tumors with mesenchymal phenotypes. This is the case for glioblastoma multiforme (GBM) (163), a devastating malignancy in which cFN was shown to be expressed in both blood vessels and tumor cells (164). In addition to cell-autonomous effects of cFN on the invasive behavior of tumor cells, paracrine effects of GBM cell-derived FN enhance the recruitment of blood vessels through integrin-dependent binding to endothelial cells.

Effects of FN on endothelial cell adhesion, spreading and migration have been extensively studied *in vitro*. However, it is important to consider that beyond its role as ligand for signaling receptors on endothelial cells, FN in perivascular matrices constitutes an obligate scaffold for organization of the vessel-associated ECM and a repository for pro-angiogenic factors [reviewed in (63)]. FN can bind directly to (165) and modulate the function of VEGF (166), one of the most potent angiogenic factors. Moreover, it has been demonstrated that astrocytic derived FN promotes retinal angiogenesis by dual integrin-dependent and -independent functions on endothelial retinal cells, promoting filopodia adhesion or VEGF-induced directional tip cell migration, respectively (167). Thus, there is still much work to be done on several fronts to fully grasp the role of FN in tumor angiogenesis and how this is linked to tumor expansion, as discussed below.

### Activating Invasion and Metastasis

Elevated FN expression is associated with invasive tumors and poor prognosis in many cancers [as reviewed in (55, 150, 163, 168–172), to cite a few]. However, this is not the case in all tumor pathologies and the role of FN in tumor invasion and metastasis has been controversial [(86) and references therein]. Lin and colleagues recently analyzed 72 studies published over the past four decades that address the role of cancer cell-derived FN (termed cancerous FN) and stromal FN in tumor progression (86). Interestingly, a tumor-suppressive function for cancerous FN was reported in 57.7% of the articles prior to 2000, yet only 15% since that date. Conversely, reports of a tumor- and metastasis-promoting role for cancerous FN increased from 11.5% before 2000 to 25% after 2000. Publications describing the implication of stromal FN in early tumor progression, but not late metastasis, remained constant at 30%.

These results raise the question of how FN came to be pro-tumoral. The increase in tumor-promoting effects of FN observed over the past several years is likely due to improved biological tools and approaches as well as a more holistic view of cancer. Indeed, since 2000 the field has evolved from the study of 2D cultured tumor lines and xenografts in immunocompromised mice models, to multimodal analysis of human tumors in their tissue context. The emergence of single cell transcriptomics (vs. transcriptomic studies that measure tumors in bulk) has refined the molecular signatures of tumor cells and different stromal cell populations thus providing a closer look at FN expression and function in different cell types, and across cancer types. Moreover, former studies on tumors were largely centered on malignant cells. However, in most carcinomas FN is produced and assembled by stromal cells (173). Indeed, recent studies have shown that stromal FN participates not only in the early steps of tumor formation but also in the promotion of tumor cell motility and invasive behavior. Remodeling the matrix by CAFs is a key feature of cancer cell invasion (174). The ability of fibroblasts to induce cancer cell invasion was found to depend on the amount of FN that they produce and assemble (175). Assembly of the protein is required, as addition of soluble FN failed to promote invasion of the cancer cells through collagen gels. Once aligned by CAFs, linear arrays of FN fibers can promote directional migration of carcinoma cells (55, 176). In the case of head and neck squamous cell carcinomas, migration of tumor cell cohorts on fibroblast-derived matrices involves the cFN-binding integrin αvβ6 and is associated with the activation of latent TGF-β at the tumor-stroma interface (55, 177). Treatment of lung tumor cells with soluble FN stimulated migration and invasion via FAK/Src/PI3K/ERK as well as activation of MMP expression (178). Thus, FN can act both as a physical scaffold laying the path for tumor cell invasion, a platform for latent TGF-β activation and a ligand for activation of intra-cellular signaling pathways and subsequent induction of matrix-degrading proteases. Finally, given that FN is an exquisitely extensible molecule, tensile forces and FN-dependent mechano-signaling in the TME play a decisive role in invasion and metastasis. The topic of ECM stiffness and tissue mechanics is excellently reviewed in (89).

#### More Than a Marker of Epithelial to Mesenchymal Transition (EMT)

FN is a mesenchymal marker par excellence. In mesenchymal-like tumors, such as glioblastomas, FN expression and assembly by tumor cells has been shown to facilitate intercellular cohesion and collective invasion through a basement membrane-like ECM (164). FN knock-down in glioma xenografts reduced tumor growth and improved survival of implanted animals (164, 179). In epithelial tumors its expression is often used to detect mesenchymal transition (180). EMT is a complex program whereby epithelial cells loose polarity and cell-cell adhesion to acquire a mesenchymal phenotype and invasive properties [reviewed in (25, 180, 181)]. Early studies described the role of FN in EMT during chick embryo gastrulation and neurulation (182). Later, FN was associated with EMT during tumorigenesis. However, it is more than just a marker of EMT. FN can contribute to mesenchymal transition by providing a platform for integrin-dependent activation of latent TGF-β (183). Thus, at the leading edge of invasive tumors paracrine interactions between CAFs and malignant epithelial cells can promote a so-called partial EMT (pEMT) phenotype characterized by the expression of EMT-related genes in tumor cells that retain their epithelial phenotype (12). This is the case for leading edge tumor cells that express αvβ6, a cellular receptor for EDA-containing cFN. In ovarian cancer, TGF-β produced by tumor cells stimulates mesenchymal transition in mesothelial cells resulting in the upregulation of FN in the ECM of mesothelial cells which increases adhesion and invasion of the sub-mesothelial basement membrane by ovarian carcinoma cells (153).

#### FN in Metastasis

Circulating FN also contributes to tumor angiogenesis and metastatic spread of malignant cells. In pFN-deficient mice, obtained by conditional KO of the *Fn1* gene in the liver, von Au and colleagues showed that a decrease in pFN reduces tumor angiogenesis, tumor growth and bone metastasis through an apparent feed forward upregulation of its own production and by modulating the response to VEGF (148). Plasma FN is one of the most abundant adhesion proteins in the blood. However, it is functionally invisible to the apical surface of endothelial cells in mature blood vessels. Following injury or angiogenic stimulation, endothelial cells upregulate cFN production and become responsive to the pro-adhesive and integrin-mediated angiogenic functions of FN. In a study by Barbazan et al., FN deposits were detected on the luminal side of hepatic blood vessels in human colorectal cancer patients (184). Using a mouse model of intestinal tumor metastasis, they demonstrated that FN deposits in the hepatic vasculature facilitate the arrest of circulating tumor cells and extravasation via a mechanism involving talin-dependent integrin signaling in the tumor cells. pFN can also promote lung metastasis by forming pFN-fibrin clots that retain circulating tumor cells via integrin αvβ3 (177).

The final step of cancer progression is colonization of secondary organs. This highly rate-limiting step is critically affected by the matrix microenvironment (185). For making a hospitable home, cancer cells need to prepare the local microenvironment before they arrive at the distant secondary site referred to as the pre-metastatic niche (186). Pioneering studies from David Lyden's lab revealed the importance of bone marrow-derived cells (BMDC) in the establishment of a pre-metastatic niche for tumor cell metastasis (187). In response to soluble factors, such as VEGF or PDGF, VEGFR1-positive BMDCs are mobilized to colonize sites of future metastasis, prior to the arrival of tumor cells, by interacting with tumor-induced EDA-containing FN through α4β1 integrin. In turn, VEGFR1-positive BMDCs secrete chemokines, such as SDF1 to attract CXCR4^+^ tumor cells to the newly formed metastatic niche. EDA-containing FN, together with tenascin C, versican and periostin have also been found in other secondary sites prior to tumor cell arrival, and may be important for recruitment of stromal cells as well as for circulating tumor cells to the pre-metastatic niche (153, 188–190).

### Avoiding Immune Destruction

#### Tumor Promoting Inflammation

The acquisition of functional capabilities allowing survival, proliferation and spread of cancer cells, defined as hallmarks most recently by Hanahan and Weinberg (142), are rendered possible by so-called enabling characteristics. One important enabling feature that has drawn much attention over the past 20 years is the inflammatory state of tumor lesions. Chronic inflammation driven by infiltrating immune cells can empower multiple cancer hallmarks.

ECM remodeling in the tumor stroma is associated to the release of proteolytic fragments, termed “matrikines,” into the microenvironment. Some of these ECM domains retain secondary structure and can display bioactivity (191) as Damage-Associated Molecular Pattern (DAMP) molecules, endogenous activators of innate immunity (192). Toll-like receptor 4 (TLR4), a DAMP receptor initially thought to be restricted to immune cells, is present and functional on a variety of non-immune normal cells and tumor cells. TLR4 has been implicated in the development of several types of cancer and fibrosis (193, 194). As part of the anti-tumor immune response, DAMP-induced TLR4 activation triggers the production of pro-inflammatory cytokines, chemokines, and effector molecules. However, continuous TLR4 signaling results in chronic inflammation. Recombinant fragments of FN containing the EDA domain, but not the full length (soluble) protein were shown to bind and activate the TLR4 (137). Binding of EDA results in TLR4-mediated NF-κB pathway activation and subsequent production of pro-inflammatory, pro-fibrotic cytokines and MMPs (137, 195, 196).

In mesenchymal cells, TLR4 signaling leads to the stimulation of a pro-fibrotic gene program with augmented expression of tissue repair, wound healing and ECM remodeling genes, while induction of inflammatory genes is relatively weak (193). A second FN Type III domain, FNIII-1c, was shown to activate TLR4-mediated inflammatory cytokine release in human fibroblasts in synergy with the EDA domain (197). Type III domains of FN can be released from the matrix by proteolysis, or become exposed in response to mechanical forces (198). Therefore, the presence of EDA-containing cFN fragments or mechanically strained fibers in the tumor matrix landscape can trigger and sustain innate immunity, inflammation, and myofibroblast generation driven by one or more EDA-dependent inflammatory feedback loops (199).

#### Regulation of Anti-tumoral Immunity

The immune system is an important barrier against tumor progression. How tumor cells develop immune system-evading mechanisms and how the different immune cells interact with tumor cells is a field of intense research. In general, the presence of tumor infiltrating CD8-expressing lymphocytes in the TME is associated with an improved prognosis and a better response to therapy in a broad range of tumor types (200–205). However, the presence of immune cells with inhibitory function, such as myeloid-derived suppressor cells and regulatory T cells (Tregs) that dampen the immune control of cancer, can be associated with worse outcome [(204) and references therein]. Analysis of histological sections of tumor samples led to the segregation of cancer immune phenotypes into three distinct profiles: immune-inflamed, immune-dessert and immune-excluded (206). Immune-inflamed and immune-dessert phenotypes are generally characterized by an abundant or sparse immune infiltrate, respectively. In immune-inflamed tumors, the immune cells are positioned in proximity to the tumor cells. Immune-excluded tumors also display an abundant infiltrate but the immune cells fail to effectively penetrate the tumor parenchyma and they remain in the stroma surrounding tumor cell nests. This immune phenotype is characterized by an excessive deposition of ECM components, including dense aligned bundles of collagen and FN around tumor islets. Live-cell imaging studies on patient-derived lung tumor tissue sections revealed active T cell motility in regions of loose FN and collagen I, whereas T cells migrated poorly in dense matrix areas surrounding tumor nests (207, 208). Thus, the ECM can promote tumor evasion from the immune system by limiting the anti-tumor activity of T cells, either directly by inhibiting the contact of infiltrating immune cells with cancer cell nests (207, 209) or indirectly through the recruitment of TAMs that cause lymphocyte retention in the stroma (210). The latter mechanism, using live imaging techniques in a mouse model and on fresh human carcinoma slices, demonstrated that TAMs impede CD8-expressing T cells from reaching tumor cells by lymphocyte trapping in the stroma and consequently limit the efficacy of immune check point inhibitor (anti-PD-1) treatment. TAMs are key components of the tumor ECM microenvironment directly affecting its production and remodeling. TAMs isolated from human ovarian carcinomas (15) and from an orthotopic colorectal cancer model (16) display a gene expression profile in which matrix glycoproteins, including FN, are highly upregulated (211). Clearly, the ECM is emerging as an important component of stromal-based immunomodulatory mechanisms that alter the trafficking, maturation and function of immune cells through multiple mechanisms, many of which have yet to be uncovered. cFN is a prominent component as a provider of DAMPs, by virtue of its obligate role in activation of ECM-tethered latent TGF-β and its function as a mechanically-tuned repository of immunomodulatory cytokines and growth factors, as discussed in previous sections.

### Deregulating Cellular Energetics

The metabolic shift toward aerobic glycolysis in cancer cells is a well-established phenomenon that is currently used in tumor diagnosis. During the past decade, numerous studies underlined a potential metabolic crosstalk between normal and transformed cells in the tumor niche (212). The link between the ECM and cell metabolic activity presents an emerging, compelling field of scientific research. Recently, several groups have provided evidence pinpointing toward shifts in cell metabolic processes mediated by matrix composition, stiffness, and remodeling (213–215). Moreover, physical properties of the TME, like pH regulation, are shaped by increased expression of pumps and transporters, and their transportation to the plasma membrane [reviewed in (216)]. Thus, the tumor landscape is sculpted and primed in order to promote cancer cell growth, motility and invasion. To our knowledge, no EDB- or EDA-oriented studies regarding cellular energetics have been reported. The handful of studies describing the role of FN Extra Domains in regulating metabolic processes has only been performed in non-tumoral contexts, thus whether the presence of the Extra Domains reflects reprogramming of cell energetics is an open question.

In an *in vivo* study, investigators used a diabetes-impaired endothelial vasodilation mouse model to study the effect of EDA-containing FN. Mice constitutively excluding EDA displayed increased endothelial dysfunction, and the underlying mechanism involved increased superoxide anion levels, NADPH oxidase (NOX4) expression, and TGF-β, suggesting a protective role of FN-EDA against vascular oxidative stress (217). Conversely, overexpression of FN-EDA or pFN in a mouse monocyte macrophage cell line, dysregulated the endogenous sterol response pathway through ER stress response, though no difference was observed between cells expressing the different isoforms (218). EDB was not included in the study, and its potential involvement is thought provoking and constitutes an interesting perspective.

## Targeting the Dysregulated Tumor ECM

### Potential Therapeutic Implications of FN

The striking implication of tumor ECM components and their cellular receptors in tumor progression and response to conventional and emerging therapies has led to the quest for novel TME-directed therapeutic strategies. Different approaches have been employed to target FN, or its receptors (219). The first approach pioneered by Neri et al. is based on antibody-mediated delivery of therapeutic agents to cFN isoforms present in the TME. Both EDA and EDB domains have been used for specific delivery of cytokines, cytotoxic agents, chemotherapy drugs and imaging agents to tumors expressing cFN variants [reviewed in (161, 220)]. The tumor-targeting immunocytokine L19-IL2 is a good example. It is composed of the human scFv antibody fragment (L19), specific to the EDB domain of FN, fused to recombinant human IL2. Following extensive preclinical studies, L19-IL2 immunocytokine has demonstrated therapeutic activity in advanced solid cancer (221). In combination with darcabazine, it displayed encouraging clinical activity in patients with metastatic melanoma in a phase I and II studies (222, 223). A randomized Phase IIb study was completed but not yet reported (NCT01055522). L19-IL2 was also tested in combination with radiotherapy. In a preclinical study, that combination enhance the radiotherapy-induced antitumor immune reaction and provided a long-lasting antitumor effect dependent on EDB expression and infiltration of cytotoxic T cells (224). A Phase I clinical study combining L19-IL2 with Stereotactic Ablative Body Radiotherapy (SABR) in oligometastic tumors (NCT02086721) was completed but results are not published. Additional clinical trials involving L19-derived targeting agents (L19-IL2, L19-TNF) alone or combined with other therapies are ongoing in several tumor types (see the NIH website identifier: https://clinicaltrials.gov).

EDA and EDB based vaccines appear to be promising for the treatment and prevention of cancer. Regarding the EDA domain, a fusion protein between streptavidin and the endogenous TLR4 ligand EDA showed the capacity to target biotinylated antigens to dendritic cells and induce T cell responses *in vivo* (225). As EDA is known to activate the NF-κB pathway leading to the activation of innate immune system and release of inflammatory cytokines, it has been explored as a cancer vaccine adjuvant in mouse models (195, 226, 227). Immunological activity of EDA depends upon its local intramolecular context within the FN chain. Immobilizing EDA-containing FN fragments within a fibrin matrix model along with antigenic peptides stimulates cytotoxic CD8^+^ T cell responses in two murine cancer models (228). Thus, delivering ECM-bound FN EDA fragments in combination with antigens could be an attractive option for anti-tumoral immunotherapies.

The second approach consists of directly interrupting the pro-tumoral effects of FN by using antibodies and small-molecule inhibitors that interrupt interactions between FN and its integrin partners. Integrins as therapeutic targets have been a focus of drug development for over 3 decades, with some successes in preclinical studies in cancer. Developments in integrin-directed therapeutics in cancer and other pathologies have been reviewed in Raab-Westphal et al. (2). Integrin α5β1, the “prototype” FN receptor, is implicated in different aspects of tumor progression, and it appears particularly overexpressed in the most aggressive tumor grades. It is a pertinent therapeutic target in solid tumors and appears safe for the patients in a phase I clinical study (229). Unfortunately, in a phase II clinical study, anti-α5β1 integrin antibody volociximab tested as monotherapy in patients with platinum-resistant advanced epithelial ovarian or primary peritoneal cancer, showed insufficient clinical activity (230). In patients with refractory or relapsed metastatic clear cell renal carcinoma, volociximab led to stable decrease in 80% of patients but no randomized controlled trial has been reported so far. Integrin α4β1, a FN receptor that binds to the EDA domain and variable region of the protein, promotes the homing of monocytes to tumors, and is essential for the participation of myeloid cells in angiogenesis and tumor growth. Specific antagonists of integrin α4β1 prevented monocyte stimulation of angiogenesis *in vivo*, macrophage colonization of tumors, and tumor angiogenesis (231). However, whereas suppression of myeloid cell homing to tumors using α4β1 antagonists appears to be an effective approach to impede tumor angiogenesis and growth, depletion of the integrin in a mouse model of colon adenocarcinoma resulted in an age-dependent effect and accelerated tumor growth in mature mice (232). These findings support a central role for α4β1 in tumor growth control but call for more in depth studies of its cellular expression pattern and function in the TME prior to its use as a pharmacological target.

The αv integrins represent an interesting class of adhesion receptors that recognize RGD-containing ligands, including FN, and have multiple roles in cancer hallmarks (e.g., angiogenesis, growth and dissemination, and immunomodulation) (233). αv-based integrins are overexpressed in several tumor pathologies and their expression can be found on both tumor cells and stromal cells. In addition to being promiscuous receptors, with FN being only one of numerous ligands, the αv-based integrins have been linked to local TGF-β activation, which compounds the complexity of their effects in the TME (234).

### Impact of FN on Treatment Response

Integrin-mediated cell-ECM and in particular cell-FN interactions confer resistance to chemotherapy as well as to ionizing radiation (235–241). In an *in vitro* and *in vivo* model of human non-small lung cancer, cetuximab promoted FN expression via p38-MAPK-ATF2 signaling (237). Cell adhesion to FN enhanced tumor cell resistance to radiotherapy, and attenuated the cytotoxic and radiosensitizing effects of cetuximab (237). More recently, chemotherapy resistance in esophageal cancer cell lines increased in cells growing in a three-dimensional environment enriched in collagen and FN (242). These results illustrate that FN plays a key role in the response of cancer cells to treatment. Conversely, anti-tumor therapies modify the tumor ECM environment, as exemplified by radiation-induced fibrosis (243).

## Concluding Remarks

Our understanding of stromal reprogramming in the TME has advanced at a rapid pace in recent years, driven by technological advances and the integration of massive amounts of information from different fields, spanning multiple scales. As illustrated above, cFN is recurrently a central component of the tumor stroma that contributes to several cancer hallmarks and enabling characteristics. Further understanding of cFN production, assembly and remodeling in primary tumor beds, draining lymph nodes and at metastatic sites is needed to grasp the full complexity of tumor progression and metastatic spread. This knowledge should provide valuable insights into cell-ECM interactions and the physical and functional interplay between different cellular components of the TME. Elucidating the role of cFN variants in modulation of immune cell trafficking, phenotype and functional maturation is of particular importance for improving the current armatorium of immunomodulatory agents and for providing therapeutic alternatives that target the stroma.

## Author Contributions

All authors listed have made a substantial, direct and intellectual contribution to the work, and approved it for publication.

## Conflict of Interest

The authors declare that the research was conducted in the absence of any commercial or financial relationships that could be construed as a potential conflict of interest.
